# Changes of gut microbiota structure in rats infected with *Toxoplasma gondii*

**DOI:** 10.3389/fcimb.2022.969832

**Published:** 2022-07-28

**Authors:** Qing-Bo Lv, He Ma, Jiaqi Wei, Yi-Feng Qin, Hong-Yu Qiu, Hong-Bo Ni, Li-Hua Yang, Hongwei Cao

**Affiliations:** ^1^ College of Life Science, Changchun Sci-Tech University, Shuangyang, China; ^2^ College of Animal Science and Veterinary Medicine, Heilongjiang Bayi Agricultural University, Daqing, China; ^3^ College of Veterinary Medicine, Qingdao Agricultural University, Qingdao, China; ^4^ School of Pharmacy, Yancheng Teachers University, Yancheng, China

**Keywords:** *Toxoplasma gondii*, fischer 344 rats, gut microbiota, 16S rRNA gene amplicon sequencing, genotype

## Abstract

*Toxoplasma gondii* (*T. gondii*) infection can cause intestinal inflammation in rodents and significantly alters the structure of gut microbiota. However, the effects of different *T. gondii* genotypes on the gut microbiota of rats remain unclear. In this study, acute and chronic *T. gondii* infection in Fischer 344 rats was induced artificially by intraperitoneal injection of tachyzoites PYS (Chinese 1 ToxoDB#9) and PRU (Type II). Fecal 16S rRNA gene amplicon sequencing was employed to analyze the gut microbiota structure at different stages of infection, and to compare the effects of infection by two *T. gondii* genotypes. Our results suggested that the infection led to structural changes of gut microbiota in rats. At the acute infection stage, the microbiota diversity increased, while both diversity and abundance of beneficial bacteria decreased at the chronic infection stage. The differences of microbiota structure were caused by strains of different genotypes. However, the diversity changes were consistent. This study demonstrates that the gut microbiota plays an important role in *T. gondii* infection in rats. The data will improve our understanding of the association between *T. gondii* infection and gut microbiota in rodents.

## Introduction

*Toxoplasma gondii* (*T. gondii*) is an important zoonotic parasite that inhabits in the nucleated cells of various hosts (Dubey et al., 2021). Mammals and birds can be infected by *T. gondii* through ingesting contaminated food and water (Almeria and Dubey, 2021). *T. gondii* infection can destroy the epithelial barrier of host intestinal mucosa and even cause fatal intestinal inflammation (Liesenfeld et al., 1996). After the host ingests cysts or oocysts, *T. gondii* invades cells in the small intestine and forms a transient parasitophorous vacuole (Suss-Toby et al., 1996; Sibley, 2004). In parasitophorous vacuole, they rapidly differentiate into tachyzoites and spread rapidly through the host *via* the vascular or lymphatic system (Lambert et al., 2006). Visceral organs (including heart), muscles, and nervous tissue are favorite sites of *T. gondii* (Dubey et al., 1998). During the acute stage of *T. gondii* infection, the host may represent mild self-limited symptoms. In general, the individuals that have normal immunity can destroy most of *T. gondii* in the acute infection phase (Dunay et al., 2018), and the remaining tachyzoites differentiate into bradyzoites, which exist as cysts in muscle, brain etc. These bradyzoites will remain largely hidden from the immune system (Cerutti et al., 2020). However, it has been suggested that the immune responses to *T. gondii* infection is sustained throughout the chronic infection, with specific increases of IgG and IFN-γ levels (Sturge and Yarovinsky, 2014).

The population structure of *T. gondii* consists of three major genetic types (Types I, II and III) and other region-specific clonal lineages (Howe and Sibley, 1995). Among them, the more common genotypes are Types II, III, 12, and Chinese 1 (Wang et al., 2016). Type II mainly infects humans and is widespread in Europe, North America, and Africa (Dubey et al., 2020). Types III and 12 are often found in animals. Type 12 was recently isolated from human and wild animals (Khan et al., 2011). In China, 78% of total isolated strains belonged to Chinese 1 (ToxoDB#9) (Jiang et al., 2013; Li et al., 2014; Wang et al., 2015) that caused severe diseases in animals and humans. The virulence of a genotype is usually determined by median lethal dose (LD_50_) of inbred mice. In comparison of Type II (LD_50_ of 100-1000 tachyzoites) and III (LD_50_ of 10^5^-10^6^ tachyzoites), Type I was identified to be the most virulent, with LD_100_ ranging from 1-10 tachyzoites (Sibley and Boothroyd, 1992; Su et al., 2002). Chinese 1 (ToxoDB#9) was also a highly virulent genotype, and 10-100 tachyzoites could kill mice in a few days (Hou et al., 2018).

Under normal conditions, *T. gondii* invasion can cause the cooperation of mucosal immune system with the gut microbiota for a resistance, however, the unbalanced gut microbiota and *T. gondii* infection may cause inflammation (Couturier-Maillard et al., 2018). Current studies suggest that there are complex interactions among *T. gondii*, mucosal immune system, and gut microbiota during *T. gondii* infection (Burger et al., 2018; Cervantes-Barragan et al., 2019; Snyder and Denkers, 2020). *T. gondii* can significantly alter the gut microbiota of mice during acute and chronic infection (Shao et al., 2020). Current studies mainly focus on the infections of Type II (ME49 or PRU) strains, and the effects of some highly virulent or locally-prevalent *T. gondii* strains on gut microbiota are not clear. However, mice had poor resistance to highly virulent strains and could not survive at the chronic infection stage. In contrast, rats may be suitable experimental animals that can withstand infection of highly virulent strains without death (Benedetto et al., 1996; Gao et al., 2015). Therefore, using rats as an experimental animal sheds new light on the changes of gut microbiota after highly virulent *T. gondii* infection.

To investigate the effects of *T. gondii* infection on gut microbiota in rats, the infection models were constructed with two *T. gondii* genotypes (Chinese 1 and Type II). The structural variations of rat gut microbiota were investigated by 16S rRNA amplification sequencing after infections of two *T. gondii* strains. Our results showed that *T. gondii* infection significantly altered the gut microbiota of rats, and different *T. gondii* genotypes caused different patterns of microbiota change. These results suggest that gut microbiota may play an important role in host resistance to parasite invasion.

## Materials and methods

### *T. gondii* strains and rats

PYS (Chinese 1, ToxoDB#9) and PRU (Type II) strains of *T. gondii* were used in this study. The tachyzoites were subcultured in human foreskin fibroblasts (HFF) cells and purified after 5 successive passages. Female Fischer 344 rats of aged 4-6 weeks (n = 18) were randomly divided into 3 groups (n = 6 per group): PYS group, PRU group, and control group. Rats were abdominally injected with 10^5^ tachyzoites of PYS or PRU strain suspended in 0.5 mL sterile phosphate buffered solution (PBS). The control group was inoculated with the same volume of sterile PBS without tachyzoites. All rats were kept in cages with independent ventilation system with free access to food and water. The feces of rats in all groups were collected on 0 (before infection), 7 and 40 days after infection, respectively. The feces were stored in cryopreservation tubes at -80°C until DNA extraction.

### Feces DNA extraction and sequencing

DNA was extracted from ~200 mg rat feces using E.Z.N.A ^®^ Mag-Bind Soil DNA Kit (OMEGA, USA) according to the manufacturer’s instructions. PCR amplification of the V3-V4 region of 16S rRNA was using the primers: 341F (5’ CCTACGGGNGGCWGCAG-3’) and 806R (5’ GGACTACHVGGGTWTCTAAT-3’). The reaction of PCR was carried out in the 20 μL system that contained 4 μL 5×*Taq* Buffer, 2 μL dNTPs, 0.8 μL of each primer, 0.4 μL *Taq* DNA Polymerase, 1 μL DNA template, and 11 μL ddH_2_O. PCR reaction conditions are as follows: pre-denaturation at 95°C for 30 s, and followed by 35 cycles of denaturation at 95°C for 30 s, annealing at 59°C for 30 s, and extension at 72°C for 45 s, and a final extension at 72° C for 10 min. The concentration and regional integrity of the PCR products were evaluated using the Agilent 5400 fragment analyzer system, and DNA was quantified using the Qubit3.0 DNA kit. Finally, standard procedures were used for DNA library construction, and 250 bp paired-end sequencing was performed on a NovaSeq 6000 PE250 platform.

### Analysis of sequencing data

Fastp (v0.23.0) was used for quality control of sequencing data (Chen et al., 2018). The low-quality sequences and sequences with N-bases were removed from the original data. Demultiplexed reads were processed with QIIME2 (v2022.2.0, https://qiime2.org) (Bolyen et al., 2019) pipeline and various built-in plugins. Briefly, the raw sequence data were quality control processed and trimmed using DADA2 algorithm (Callahan et al., 2016), and the primer information and chimeric sequences were removed. DADA2 was also used to de-noise reads into amplicon sequence variants (ASVs) and obtain the profile of feature sequence. ASVs with an average abundance less than 0.001% were deleted. Taxonomy was assigned to ASVs using SILVA (138.1-SSURef-NR99) database reference sequences (Quast et al., 2012).

### Statistical analyses

All statistical analyses were completed on R (v.4.1.1) platform, including diversity analysis, species composition analysis, principal component analysis, etc. Briefly, the ‘diversity’ function in the *vegan* package (Oksanen et al., 2020) was used to calculate Shannon index of samples. The Bray-Curtis distance between samples was calculated using the ‘vegdist’ function of *vegan* package; the ‘adonis’ function was used to implement permutational multivariate analysis of variance (PERMANOVA). Visualization of species composition and principal component analysis were implemented by *ggplot2* package. Wilcoxon rank sum test was used to measure differences between groups, * (*p* < 0.05), ** (*p* < 0.01), and *** (*p* < 0.001). Lefse analysis was performed using the Galaxy online platform (Afgan et al., 2018) (https://huttenhower.sph.harvard.edu/galaxy).

## Results

### *T. gondii* infection is associated with gut bacterial diversity

A total of 54 fecal samples were divided into nine groups: PYS-0d (n = 6), PYS-7d (n = 6), PYS-40d (n = 6), PRU-0d (n = 6), PRU-7d (n = 6), PRU-40d (n = 6), Control-0d (n = 6), Control-7d (n = 6), and Control-40d (n = 6). It should be noted that it was necessary to collect samples for control group from the same period. Studies have shown that the gut microbiota of young rats is dynamically, and the structure of microbiota changes with the extension of feeding time (Flemer et al., 2017). 16S rRNA gene amplicon sequencing was performed on these samples, and a total of 580.8 Mb clean data were obtained. We used DADA2 algorithm to de-noise and cluster the data, and thus generating a total of 6,207 ASVs sequences. ASVs with an average relative abundance less than 0.001% were deleted, and 1,604 ASVs were used for the subsequent analysis.

**Figure 1:**
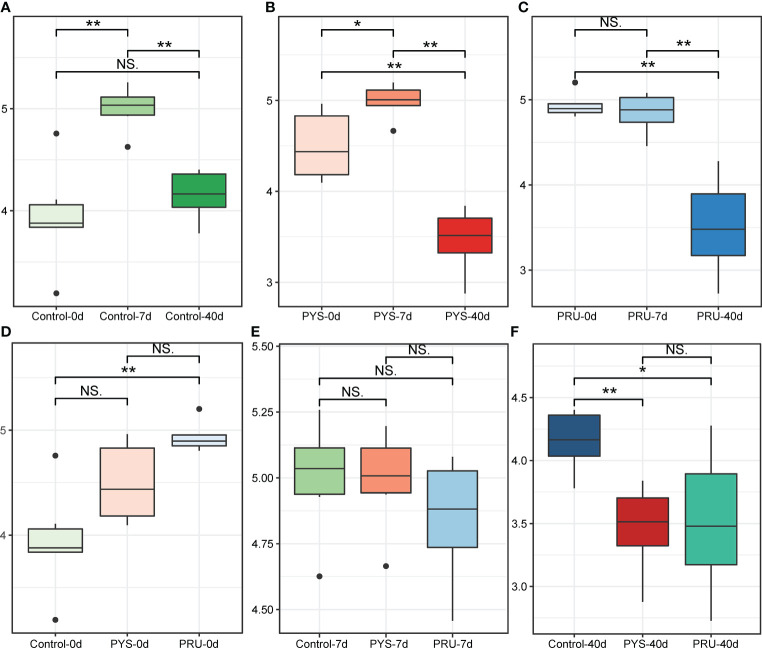
α diversity of gut microbiota in rats. **(A–C)** Shannon diversity index of control, PYS and PRU groups in different infection periods. **(D–F)** Shannon index of rats before infection, 7 and 40 days after infection, respectively. The significance level in the Wilcoxon rank sum test is denoted as: *p < 0.05 and **p < 0.01. For boxplot, boxes represent the interquartile range between the first and third quartiles and median (internal line); whiskers denote the lowest and highest values within 1.5 times range of the first and third quartiles, respectively; and nodes represent outliers beyond the whiskers. NS means "Not significant".

To assess diversity changes after *T. gondii* infection, the Shannon index was calculated for each group. We first assessed changes in gut diversity in normal rats. Interestingly, since the beginning of the experiment, the diversity of gut microbiota of rats showed a law of first increasing and then decreasing with the extension of feeding time (**Figure 1A**). This was consistent with the phenomenon reported in a previous study (Flemer et al., 2017). Next, the diversity of gut microbiota in the infected rats was assessed. We found a same pattern of diversity changes in the infected and control groups, whereas a more severe diversity decline occurred during the chronic infection stage (**Figures 1B, C**). This indicated that the chronic infection of *T. gondii* was closely related to the diversity of gut microbiota.

In addition, the changes of the diversity during the same period were further compared. It was noteworthy that the diversity of the PRU group before infection was higher than that in other groups (**Figure 1D**). However, this did not affect the comparison results between groups in the same period. After 7 days of infection, there was no significant difference in gut microbiota diversity between the infected and control rats (**Figure 1E**). After 40 days of infection, the gut microbiota diversity of the infected group was significantly lower than that of the control group (**Figure 1F**). In addition, there were no significant differences among different genotypes (**Figure 1F**).

### *T. gondii* infection results in structural changes of gut microbiota in rats

**Figure 2:**
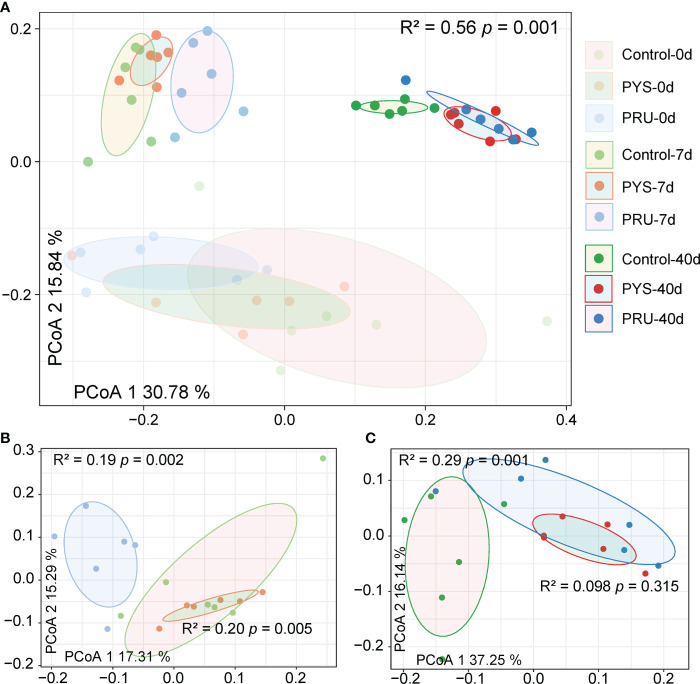
Differences in gut microbiota structure between groups. **(A)** PCoA analysis based on the Bray-Curtis distance of ASVs reveals the separation between different groups. The position of sample (represented by nodes) in the first two principal coordinates is shown below. **(B)** and **(C)** Differences between groups on day 7 and 40 post infection, respectively. R^2^ and *p* values represent the results of PERMANOVA.

Then, in order to further understand the differences of gut microbiota between groups, a principal coordinate analysis (PCoA) was conducted based on the Bray-Curtis distance. The results showed that the distance between different groups was far, with an obvious separation (**Figure 2A**). The infected and control groups in different periods were divided into three regions. The rat samples before infection were at the bottom, and samples of 7 days after infection were at the top left, and samples of 40 days after infection were at the top right. The explanatory variance of PC1 and PC2 was 30.78% and 15.84%, respectively. In addition, the PERMANOVA analysis showed significant differences among groups (R^2^ = 0.56, *p* = 0.001). PERMANOVA in the infected group showed that *T. gondii* infection of different genotypes also led to the variation of bacterial community structure. After 7 days of infection, there was a separation between PYS and PRU groups (**Figure 2B**, [R² = 0.20, *p* = 0.005]), whereas PYS and control groups were closer. After 40 days of infection, samples from the two infection groups were close to each other (**Figure 2C**, [R² = 0.098, *p* = 0.315]), but farther from the control group. These results suggested that the differences in infection process of *T. gondii* genotypes might be related to the gut microbiota.

### Comparison of gut microbiota in different infection periods

**Figure 3:**
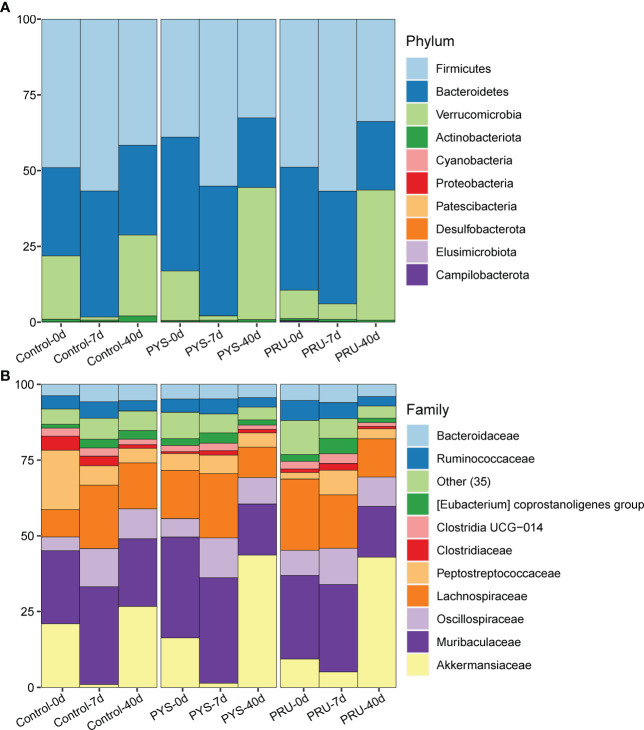
Gut microbiota structure of rats. **(A)** The gut microbiota structure at phylum level of each group. **(B)** The gut microbiota structure at the family level of each group, and only the top 10 families have the high abundance, while 35 low-abundance families are integrated into the “Other” category.

To further understand the structural variation of gut microbiota in rats infected with *T. gondii*, the differences were assessed among groups at different taxonomic classification. At the phylum level, Firmicutes, Bacteroidetes, and Verrucomicrobia were found to be the dominant flora in the intestinal tract of rat. In the control group, the abundance of Verrucomicrobia decreased with age and then returned to earlier levels. Similarly, the abundance of Verrucomicrobia decreased and then increased in the infected group. However, after 40 days of infection, the abundance levels of Verrucomicrobia in PYS and PRU groups were significantly higher than those in the uninfected and 7-day groups, accompanied with a decrease in Bacteroides and Firmicutes (**Figure 3A**, **Table S1**). At the family level, we found that Akkermansiaceae (mainly *Akkermansia*) was the main differentiator. In the control group, Akkermansiaceae decreased on day 7 and recovered to day 0 level after 40 days of infection. In PYS and PRU infected groups, after 40 days of infection, the relative abundance of Akkermansiaceae did not return to the level of 0 day as the control group, but increased significantly (**Figure 3B**, **Table S2**). In addition, compared with the control group, the relative abundance of Muribaculaceae and Lachnospiraceae in the two infection groups was significantly decreased on day 40 after infection. After 7 days of infection, Akkermansiaceae of PRU group was slightly higher than that of PYS group, while after 40 days of infection, the bacterial community structures of PRU and PYS groups were almost the same. This indicated that different genotypes had different damage to intestinal tract at the early infection stage, which might be caused by the difference in virulence of *T. gondii* strains.

### The genus-level resolution revealed the effect of *T. gondii* infection

**Figure 4:**
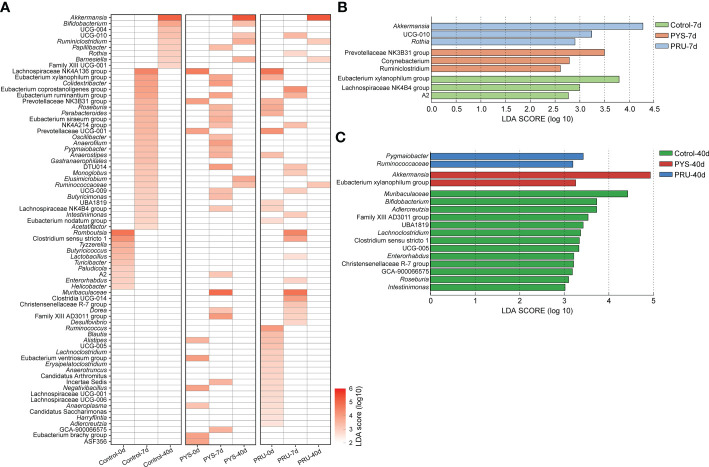
Lefse analysis of genus level. **(A)** The heat map is divided into three areas. Each area shows the different genera of control, PYS, and PRU groups in different infected periods. The red gradient represents the LDA score (log10) value. Blank means no significant difference or decline of the genus between groups. Briefly, blank means no significant difference when all three groups are blank, and enriched in one group means it is absent in the other two groups. **(B)** and **(C)** Differences at genus level between groups on day 7 and 40 post infection, respectively. The higher value of LDA Score (log10), the more significant difference is present between groups.

To look for differences between groups at the genus level, Lefse was used to identify differentially rich genera with LDA > 2.0 in each group. A total of 72 genera were significantly different between groups from day 0 to 40 of longitudinal comparison. Among them, the rats for 40 days after infection had the least number of genera, indicating that the gut microbiota in rats at chronic infection stage of *T. gondii* was out of order. To reduce the effects of changes in the normal gut microbiota, we focused on genera that showed no significant difference in the control group, but significant difference in the infected group. *Muribaculaceae*, *Dorea*, and Family_XIII_AD3011_group were significantly enriched in PYS-7d and PRU-7d groups, while *Alistipes*, Eubacterium_ventriosum_group, *Negativibacillus*, and *Anaeroplasma* were significantly absent after *T. gondii* infection (**Figure 4A**). In addition, we also found some bacteria (e.g., Prevotellaceae_NK3B31_group, Prevotellaceae_UCG-001, and Lachnospiraceae_NK4A136_group) that were opposite to the control group were enriched in Control-7d group and only few were found on days 7 and 40 in the *T. gondii* infected group.

Then, we compared the bacterial genus differences between groups in the same infection period. After 7 days of infection, Prevotellaceae_NK3B31_group, *Corynebacterium*, and *Ruminiclostridium* were significantly enriched in PYS infected group. *Akkermansia*, UCG-010, and *Rothia* were significantly enriched in PRU infected group (**Figure 4B**). In contrast, *Corynebacterium*, *Ruminiclostridium*, *Eubacterium* Xylanophilum group, Lachnospiraceae NK4B4 group, and A2 were significantly reduced in the infected group. *Akkermansia* and Eubacterium_Xylanophilum_group were significantly enriched in PYS infected group after 40 days of infection. *Pygmaiobacter* and *Ruminococcaceae* were significantly enriched in PRU infected group (**Figure 4C**). These results suggested that differences were present in gut microbiota structure among rats infected with different *T. gondii* strains. Compared with acute infection period (7 days), the loss of bacterial community was more serious in chronic infection period. There were 13 genera dropped significantly in the infection group (**Figure 4C**), such as *Muribaculaceae*, *Bifidobacterium*, *Adlercreutzia*, *Lachnoclostridium*, *Enterorhabdus*, *Roseburia*, *Intestinimonas*, etc. In brief, these results suggested that during chronic *T. gondii* infection, the gut microbiota did not return to its healthy state, or even appeared more severe decline of bacteria.

## Discussion

*T. gondii* infection can cause inflammatory responses in intermediate hosts, and the degree is related to the virulence of different genotypes (Sanchez and Besteiro, 2021). Highly virulent genotypes cause severe inflammatory responses and even death of hosts in the acute phase. The avirulent genotype causes a persistent inflammatory response, but is not fatal. Multiple studies have shown that inflammatory responses alter the structure of microbiota in rodents’ guts (Hildebrand et al., 2013; Menghini et al., 2019). Therefore, we aimed to explore the structural variation of gut microbiota in rats during the acute and chronic stages of *T. gondii* infection and analyze the differences among different genotypes.

In this study, rats were infected with high virulence strain PYS and avirulent strain PRU, respectively. The difference of gut microbiota structure between acute and chronic infection stages was observed. Our results suggested that intestinal inflammation caused by *T. gondii* infection was closely related to changes of microbiota in guts. During acute infection period, the bacteria in rat gut showed a broad increase in α diversity (**Figures 1B, C**). This result is consistent with the phenomenon observed in mice previously (Shao et al., 2020). However, we did not observe an increase in the abundance of pathogenic bacteria (e.g., Proteobacteria), which might be due to the impact of *T. gondii* infection on rats was different from that on mice. In addition, a decline in the abundance of some beneficial bacteria, such as *Alistipes*, Eubacterium_ventriosum_group and *Anaeroplasma*, at the acute infection stage (**Figure 4B**). Dysregulation of these bacteria is a typical feature of inflammation in the body, especially changes in the abundance of *Alistipes* and *Anaeroplasma* are thought to be associated with a variety of diseases (Parker et al., 2020; Liu et al., 2022).

It is of interest to note that the proportions of *Akkermansia*, *Muribaculaceae*, and *Dorea* in rats. These bacteria are common in the guts of rats, and their increase suggests that the gut microbiota is disturbed. *Akkermansia* mainly refers to *Akkermansia muciniphila* that is a kind of probiotics widely exists in the intestinal mucus layer of mammals (Zhai et al., 2019). Its probiotic effects, including metabolic regulation, immune regulation, and intestinal health protection, have been widely studied (Cani et al., 2022). A variety of diseases, including metabolic syndrome, autoimmune, and cancer, have been reported to be associated with *A. muciniphila* abundance (Olson et al., 2018; Blacher et al., 2019; Derosa et al., 2022). Yan et al. found that *A. muciniphila* significantly increased in the guts of *T. gondii* infected mice after koumiss treatment (Yan et al., 2022), thus suggesting that bacteria were involved in the prevention of *T. gondii* infection. In addition, Liu et al. reported that *A. muciniphila* abundance was elevated in the guts of mice infected with *Trichinella spiralis* (Liu et al., 2021). A recent study showed that oral supplementation of *A. muciniphila* promoted *T. spiralis* expulsion *via* TLR2 in mice (Jin et al., 2022). These reports suggest that *A. muciniphila* plays an important role in protecting guts from parasitic invasion. However, whether the increase of these bacteria in rats infected by *T. gondii* infection is related to the parasite pathogenesis or the immune regulatory function remains to be further examined.

During the chronic infection period, the bacterial diversity of rats was significantly decreased. This is probably a normal phenomenon in the growth and development of rats, similarly to a previous report (Flemer et al., 2017). However, the infected rats showed a more severe decrease in microbiota diversity than the control ones (**Figures 1B, C**). This indicates that the continuous inflammatory response caused by chronic infection of *T. gondii* may affect the normal development of gut microbiota in rats, so that the microbiota cannot be restored to the level of normal status. Compared with the acute infection period, the number of bacteria that was rich in chronic infection was significantly reduced, and the loss of bacteria was significantly increased. In addition, *Akkermansia* maintained a high abundance level at chronic stage. This may be due to a decrease in other bacteria, resulting in a higher percentage of *Akkermansia* (**Figure 3B**). Most of the missing bacteria have been reported as beneficial bacteria, such as *Bifidobacterium*, *Adlercreutzia*, etc.

It is well known that there were significant differences in pathogenicity between different virulence genotypes. Interestingly, although there were differences in the patterns of change in bacterial communities in PYS and PRU groups, the diversity changes in both groups were almost identical (**Figures 1E, F**). Thus, virulent strain PYS may not cause more severe bacterial disorder than avirulent strain PRU. This may be related to the rats’ natural resistance to *T. gondii* infection (Li et al., 2012). Limited studies have shown that some highly toxic strains, e.g., GUY008-ABE, can cause rats’ death and show a high parasite burden in multiple organs (Loeuillet et al., 2019). Dubey et al. reported the effects of *T. gondii* genotypes on parasite distribution, location, tissue cyst size, or tissue lesions (Dubey et al., 2016). However, although a considerable number of studies have assessed the pathological and immunological changes in rats infected with *T. gondii* (Tonin et al., 2013; Trevizan et al., 2016; Schneider et al., 2018; Machado et al., 2021), there is still a lack of comparison of intestinal damage caused by parasite genotypes in rats. Therefore, we have not yet understood the internal relationship between the structural changes of gut microbiota caused by different genotypes of *T. gondii* and the infection mechanism, which needs to be further studied.

Our results were in contrast with the findings by Taggart et al. (2022), who reported that the chronic infection of *T. gondii* did not change the gut microbiota structure of Wistar Hannover rats and concluded that chronical infection may not cause persistent inflammation in rats. The differences for these results may be due to a variety of experimental designs. For example, in the selection of rat strains, we carefully referred to the studies performed by Sergent et al. (2005) and Gao et al. (2015). Fischer 344 rats used in this study were more sensitive to *T. gondii* infection than Wistar and Lewis rats. This rat is closer to the clinical process of human infection and better reflects human immune resistance to *T. gondii* infection (Dubey and Frenkel, 1998; Zenner et al., 1999). On the other hand, in order to avoid gender influencing our judgment on the structural variation of gut microbiota, we only selected female rats for the experiments. In conclusion, our results extend the conclusions of previous studies and suggest that different strains of rats may influence the experimental results.

In conclusion, our study suggests that there is a close relationship between gut microbiota and *T. gondii* infection in rats. The changes in the structure of gut microbiota from the acute to chronic periods suggest a sustained inflammatory response. The comparison between PYS and PRU strains indicated that the higher virulent strain did not necessarily cause more serious imbalance of guts. However, the limitations of this study are as follows: 1) There is a lack of more assessment information on *T. gondii* genotypes, such as RH, which is more virulent; 2) Lack of quantitative evaluation of intestinal inflammation degree in rats; 3) Confounding factors, such as rat strain and hormone levels, may influence the final results. Therefore, it is necessary to introduce more genotypic, pathological, and immunological information in the future studies, and to evaluate the relationship between *T. gondii* virulence and gut microbiota through stricter variable control.

## Data availability statement

The datasets presented in this study can be found in online repositories. The names of the repository/repositories and accession number(s) can be found below: https://www.ncbi.nlm.nih.gov/bioproject/PRJNA848626/, PRJNA848626.

## Ethics statement

The animal study was reviewed and approved by Animal Ethics Committee of Qingdao Agricultural University.

## Author contributions

HW, L-HY, HM and Q-BL, conceived of the study. Q-BL, H-YQ and Y-FQ, completed the experiment, data analysis and writing section of this study. HM, H-BN and JW, contributed to revising manuscript. All authors read and approved the final manuscript.

## Funding

This work was supported by the Research Foundation for Distinguished Scholars of Qingdao Agricultural University (665-1120046).

## Acknowledgments

The author would like to thank all the members of the author’s laboratory for their great help in carrying out the work.

## Conflict of interest

The authors declare that the research was conducted in the absence of any commercial or financial relationships that could be construed as a potential conflict of interest.

## Publisher’s note

All claims expressed in this article are solely those of the authors and do not necessarily represent those of their affiliated organizations, or those of the publisher, the editors and the reviewers. Any product that may be evaluated in this article, or claim that may be made by its manufacturer, is not guaranteed or endorsed by the publisher.
